# Amino Acid Composition of an Organic Brown Rice Protein Concentrate and Isolate Compared to Soy and Whey Concentrates and Isolates

**DOI:** 10.3390/foods3030394

**Published:** 2014-06-30

**Authors:** Douglas S. Kalman

**Affiliations:** Nutrition/Endocrinology Department, Miami Research Associates, 6141 Sunset Drive, Suite 301, Miami, FL 33143, USA; E-Mail: dkalman@miamiresearch.com; Tel.: +1-305-666-2368; Fax: +1-305-595-9239

**Keywords:** brown rice, supplemental protein, essential amino acids, branched-chain amino acids, muscle protein synthesis

## Abstract

A protein concentrate (Oryzatein-80™) and a protein isolate (Oryzatein-90™) from organic whole-grain brown rice were analyzed for their amino acid composition. Two samples from different batches of Oryzatein-90™ and one sample of Oryzatein-80™ were provided by Axiom Foods (Los Angeles, CA, USA). Preparation and analysis was carried out by Covance Laboratories (Madison, WI, USA). After hydrolysis in 6-N hydrochloric acid for 24 h at approximately 110 °C and further chemical stabilization, samples were analyzed by HPLC after pre-injection derivitization. Total amino acid content of both the isolate and the concentrate was approximately 78% by weight with 36% essential amino acids and 18% branched-chain amino acids. These results are similar to the profiles of raw and cooked brown rice except in the case of glutamic acid which was 3% lower in the isolate and concentrate. The amino acid content and profile of the Oryzatein-90™ isolate was similar to published values for soy protein isolate but the total, essential, and branched-chain amino acid content of whey protein isolate was 20%, 39% and 33% greater, respectively, than that of Oryzatein-90™. These results provide a valuable addition to the nutrient database of protein isolates and concentrates from cereal grains.

## 1. Introduction

Concentrated protein powders, commonly used as dietary supplements and in food processing, are available in a variety of flavors and forms, including ready to drink shakes, bars, bites, oats, gels and powders manufactured using soy, milk, peas, or eggs as the source of the protein. Protein concentrates are created by pushing the protein source through a very small filter that allows water, minerals, and other organic materials to pass though. The proteins, which are too big to pass through the filter, are collected, resulting in protein powder or protein concentrate. Concentrates can have substantial amounts of carbohydrate and fat. Further purification using additional filtration or a technique called ion-exchange or cross-flow microfiltration [1,2,3]. Results in the formation of the protein isolate. Isolates have very low levels of carbohydrates and fat and are almost exclusively pure protein.

Soy protein concentrate is made from defatted soy flour without the water-soluble carbohydrates but with the fiber. It contains approximately 70% protein. Soy protein isolate is a more purified form of soy protein which has had most of the non-protein elements removed. It is almost 90% pure protein and has a very neutral flavor. Soy protein concentrates and isolates have long been used for a variety of food processing purposes such as increasing the protein content of foods, enhancing moisture retention, and acting as emulsifiers. 

Whey protein concentrate contains 70%–85% protein and up to 5% lactose. People with lactose intolerance may experience gastric discomfort when consuming large amounts of whey protein concentrates, a problem that is eliminated with whey protein isolates. Whey protein isolate is rapidly digested making it a popular supplement among athletes attempting to rebuild muscle after a strenuous workout [4,5]. 

The amino acid composition of protein isolates has made them popular as dietary supplements. Although the concentration of sulfur-containing amino acids is low in soy protein isolates, soy contains a high concentration of branched-chain amino acids (BCAA). BCAA are concentrated in muscle tissue and used to fuel working muscles and stimulate protein synthesis [6,7]. Whey proteins are highly bioavailable, very quickly absorbed into the body, and also have a high concentration of BCAA. Moreover, whey protein isolate is an excellent source of lysine which is often the rate-limiting amino acid in grains and legumes [8]. Like most Americans, athletes consume more than enough dietary protein, questioning the need for protein shakes. However, some evidence supports the idea that protein shakes are superior to whole foods with regard to enhancing muscle hypertrophy in the one hour window following intensive exercise [9].

Brown rice is an excellent source of protein, containing 37% of the total protein as essential amino acids and 18% as BCAA ([10], item 20036). We are not aware of a comprehensive analysis of the amino acid profiles of a brown rice protein concentrate or isolate. Thus the purpose of the present study was to compare the amino acid profile of an organic brown rice protein concentrate (Oryzatein Ultra Silk-80, Axiom Foods, Los Angeles, CA, USA) and a brown rice protein isolate (Oryzatein Ultra Silk-90, Axiom Foods, Los Angeles, CA, USA) to that of published standards for raw and cooked brown rice, soy protein isolate and concentrate, and whey protein isolate and concentrate.

## 2. Materials and Methods

### 2.1. Materials

Commercially available rice protein isolate and concentrate as prepared and sold by the manufacturer (Axiom Foods/Growing Naturals, Los Angeles, CA, USA) were analyzed for their amino acid content by Covance Laboratories (Madison, WI, USA). The samples included Organic Oryzatein Ultra 90 isolate, Batch # HZN11008-13 (received 15 February 2012) Organic Oryzatein Silk 80 concentrate, Batch # HZN11018-22 (12 March 2012) and Organic Oryzatein Silk 90 isolate, Batch # HZN12004-111 (received 15 May 2012). The accompanying paperwork to the analytical laboratory indicated that the serving size for each was ~34 g. As the products were already in powder form, no further preparation was performed prior to analysis. The products were stored at minus 20 °C until the start of analysis, which took place approximately one week after receipt of each sample. 

### 2.2. Preparation and HPLC Analysis of Amino Acid in Rice Protein Isolate and Concentrate

Prior to analysis, all samples were mixed following Covance SOP NA-FDA-156 v.1, which outlines inspecting the sample to ensure it appears homogeneous and also mixing the sample prior to weighing. Sample sizes of ~0.1 g were hydrolyzed in 40 mL 6-N hydrochloric acid for 24 h at approximately 110 °C. An appropriate amount of phenol was added to prevent halogenation of tyrosine. Cystine and cysteine were converted to *S*-2-carboxy-ethylthiocysteine by the addition of dithiodipropionic acid and hydrolyzed for 24 h in 6-N HCl. Tryptophan was hydrolyzed from proteins (using a ~0.2 g sample size) by heating at approximately 110 °C in 4.2-N NaOH (for 20 h). Samples were analyzed by high performance liquid chromatography (HPLC) after pre-injection derivitization [3]. The primary amino acids were derivitized with *O*-phthalaldehyde (OPA) and the secondary amino acids were derivitized with fluorenylmethyl chloroformate (FMOC) before injection [1,2,3]. The isolate was further purified by ion-exchange or cross-flow microfiltration [3]. The result of the analysis of Oryzatein-90 represents the average of the two analyses.

The amino acid profiles of Oryzatein-80 and Oryzatein-90 were compared to the published amino acid profiles for raw ([10], item 20036) and cooked brown rice ([10], item 20037), soy protein isolate ([10], item 16122) and concentrate ([10], item 16420), and whey protein concentrate and isolate [11].

## 3. Results and Discussion

### 3.1. Total Amino Acid Contents of Brown Rice Protein Isolate and Concentration

The results of the present study indicate that an organic brown rice protein concentrate, Oryzatein-80 and its isolate, Oryzatein-90 are excellent sources of total, essential, and branched-chain amino acids and are comparable to soy and whey protein concentrates and isolates. The total amino acid (TAA) content of both the brown rice concentrate and isolate were approximately 78% by weight with 36% essential amino acids (EAA) and 18% branched-chain amino acids (BCAA) (Table 1). These profiles were very similar to those of cooked brown rice (Table 1) and raw brown rice (data not shown) except in the case of glutamic acid, which was 3% lower in the isolate and concentrate. Cooking had no effect on the amino acid profile of brown rice.

### 3.2. Comparison and Variation of Amino Acid Concentration between Sources of Brown Rice, Whey, and Soy

**Figure 1:**
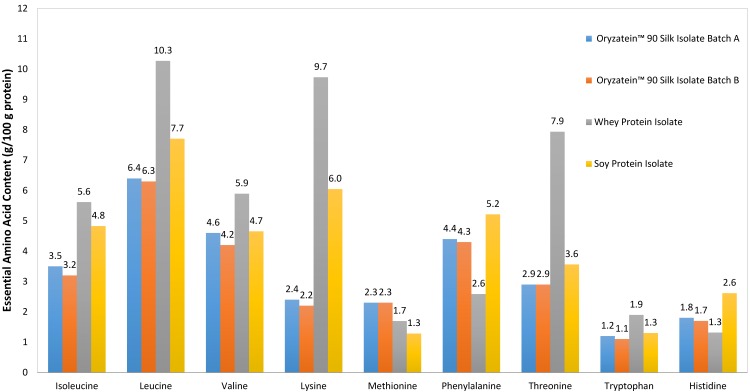
Essential amino acid profiles of Oryzatein-90 organic brown rice batch A and B, soy, and whey protein isolates.

Oryzatein-90 isolate contains 79% TAA by weight, soy protein isolate is 88% TAA by weight, and whey protein isolate is almost 100% amino acids (Table 2). The amino acid profile and content of soy protein isolate was similar to that of Oryzatein-90; both contain >36% EAA and >17% BCAA (Table 2). Although Oryzatein-90 was a better source of valine and methionine, soy protein isolate was a better source of lysine. Whey protein isolate contains 46% EAA and 22% BCAA (Figure 1) and is a richer source of isoleucine, leucine, lysine and threonine but not phenylalanine compared to Oryzatein-90 (Figure 1).

Oryzatein-80 brown rice concentrate contains 77% TAA by weight, 36% EAA and 18% BCAA, similar to the isolate (Table 1). The amino acid profiles of the soy and whey protein concentrates were also similar to their isolates. Although soy protein concentrate has 63% TAA, the percent of EAA and BCAA is similar to that of Oryzatein-80 (Table 3). Whey protein concentrate, similar to the isolate, contains 99% TAA, 53% EAA, and 24% BCAA (Table 3).

The amino acid concentrations of the various isolates and concentrates all differ from the amino acid concentrations of their respective source materials, namely raw brown rice, defatted soy flour, and cow’s milk. The concentrations of lysine and glutamate decrease but those of methionine, tyrosine and cystine increase in the brown rice protein isolate Oryzatein-90 compared to raw brown rice (Table 1). In contrast, there are few differences in the amino acid profiles of soy protein isolate and defatted soy flour (data not shown). Whey protein isolate has lower concentrations of phenylalanine, proline and glutamate but higher concentrations of threonine, alanine and aspartate compared to cow’s milk (data not shown).

Although not addressed in this analysis and report, it appears that Oryzatein-90 may have utility over soy and whey protein isolates for several reasons: it is not genetically modified; does not contain lactose; and does not come from an animal known to be treated with growth hormones, anabolic steroids, estrogens and other hormones, antibiotics or other chemicals that may have an impact upon human health.

Moreover, a recent study comparing the effects of rice or whey protein isolate in male athletes immediately post exercise found no differences in perceived recovery or soreness between the two isolates [12].

**Table 1:** Amino acid composition of Oryzatein Silk 90 brown rice protein isolate, Oryzatein Silk 80 brown rice protein concentrate and cooked *Oryza sativa* L. (brown rice).

Amino Acid	Oryzatein 90 Silk Isolate				Oryzatein		Oryza sativa	
	Batch A ^a^		Batch B ^b^		80 Silk Concentrate ^c^		Cooked ^d^	
	mg/100 g	% Total AA	mg/100 g	% Total AA	mg/100 g	% Total AA	mg/100 g	% Total AA
Alanine	4469	5.8	4381	5.7	4322	5.6	151	5.8
Arginine	6321	8.2	6350	8.3	6115	7.9	196	7.6
Aspartic acid	6938	9.0	6791	8.9	6762	8.7	242	9.4
Cystine	1697	2.2	1808	2.4	1629	2.1	31	1.2
Glutamic acid	13,906	18.0	13,700	17.9	13,495	17.4	526	20.4
Glycine	3528	4.6	3410	4.5	3410	4.4	127	4.9
Histidine	1820	2.4	1670	2.2	1699	2.2	66	2.6
Isoleucine	3469	4.5	3234	4.2	3381	4.4	109	4.2
Leucine	6409	8.3	6321	8.3	6203	8.0	214	8.3
Lysine	2420	3.1	2190	2.9	2120	2.7	99	3.8
Methionine	2270	2.9	2258	3.0	2279	2.9	58	2.2
Phenylalanine	4410	5.7	4292	5.6	4116	5.3	133	5.1
Proline	2881	3.7	3675	4.8	3557	4.6	121	4.7
Serine	3910	5.1	3881	5.1	3793	4.9	134	5.2
Threonine	2919	3.8	2858	3.7	2799	3.6	95	3.7
Tryptophan	1170	1.5	1150	1.5	1120	1.4	33	1.3
Tyrosine	4263	5.5	4263	5.6	6203	8.0	97	3.8
Valine	4557	5.9	4263	5.6	4469	5.8	151	5.8
Total amino acids	77,357	100.0	76,495	100.0	77,472	100.0	2583	100.0
Total EAA/% total	29,444	38.1	66,940	36.9	28,186	36.4	958	37.1
Total BCAA/% total	14,435	18.7	13,818	18.1	14,053	18.1	474	18.4

^a^ Results of Covance^®^ Laboratories Inc. (Madison, WI, USA) Axiom Foods Organic Oryzatein Ultra 90 Batch HZN11008-13. Sample: 120021. Certificate of analysis. Report 518291-0. Received on 17 February 2012. ^b^ Results of Covance^®^ Laboratories Inc. Axiom Foods Organic Oryzatein Ultra 90 Batch No. HZN12004-111. Sample 1360846. Certificate of analysis. Report 570345-0. Received 24 May 2012. ^c^ Results of Covance^®^ Laboratories Inc. Axiom Foods Organic Oryzatein Ultra 80 Batch HZN11018-22. Sample: 1250431. Certificate of analysis. Report 535689-0. Received on 21 March 2012. ^d^ U.S. Department of Agriculture, agricultural research service. 2013. USDA National Nutrient Database for Standard Reference, Release 26 [10].

**Table 2:** Amino acid composition of Oryzatein brown rice protein isolate, whey protein isolate and soy protein isolate.

Amino Acid	Oryzatein 90 Silk Isolate				Soy Protein		Whey Protein	
	Batch A ^a^		Batch B ^b^		Isolate ^c^		Isolate ^d^	
	mg/100 g	% Total AA	mg/100 g	% Total AA	mg/100 g	% Total AA	mg/100 g	% Total AA
Alanine	4469	5.8	4381	5.7	3589	4.1	4800	4.8
Arginine	6321	8.2	6350	8.3	6670	7.6	1779	1.8
Aspartic acid	6938	9.0	6791	8.9	10,203	11.6	10,161	10.2
Cystine	1697	2.2	1808	2.4	1046	1.2	2089	2.1
Glutamic acid	13,906	18.0	13,700	17.9	17,452	19.8	19,311	19.4
Glycine	3528	4.6	3410	4.5	3603	4.1	1421	1.4
Histidine	1820	2.4	1670	2.2	2303	2.6	1311	1.3
Isoleucine	3469	4.5	3234	4.2	4253	4.8	5600	5.6
Leucine	6409	8.3	6321	8.3	6783	7.7	10,239	10.3
Lysine	2420	3.1	2190	2.9	5327	6.0	9700	9.7
Methionine	2270	2.9	2258	3.0	1130	1.3	1689	1.7
Phenylalanine	4410	5.7	4292	5.6	4593	5.2	2579	2.6
Proline	2881	3.7	3675	4.8	4960	5.6	5739	5.8
Serine	3910	5.1	3881	5.1	4593	5.2	4921	4.9
Threonine	2919	3.8	2858	3.7	3137	3.6	7911	7.9
Tryptophan	1170	1.5	1150	1.5	1116	1.3	1889	1.9
Tyrosine	4263	5.5	4263	5.6	3222	3.7	2679	2.7
Valine	4557	5.9	4263	5.6	4098	4.7	5879	5.9
Total amino acids	77,357	100.0	76,495	100.0	88,078	100.0	99,697	100.0
Total EAA/% total	29,444	38.1	66,940	36.9	32,740	37.2	46,797	46.9
Total BCAA/% total	14,435	18.7	13,818	18.1	15,134	17.2	21,718	21.8

^a^ Results of Covance^®^ Laboratories Inc. Axiom Foods Organic Oryzatein Ultra 90 Batch HZN11008-13. Sample: 120021. Certificate of Analysis. Report 518291-0. Received on 17 February 2012. ^b^ Results of Covance^®^ Laboratories Inc. Axiom Foods Organic Oryzatein Ultra 90 Batch No. HZN12004-111. Sample 1360846. Certificate of Analysis. Report 570345-0. Received 24 May 2012. ^c^ U.S. Department of Agriculture, Agricultural Research Service. 2013. USDA National Nutrient Database for Standard Reference, Release 26, item 16122 [10]. ^d^ US Dairy Export Council. 2004. Applications Monograph. Senior Nutrition [11].

**Table 3:** Amino Acid Composition of Oryzatein Brown Rice Protein Concentrate, Whey Protein Concentrate and Soy Protein Concentrate.

Amino Acid	Oryzatein		Soy Protein		Whey Protein	
	80 Silk Concentrate ^a^		Concentrate ^b^		Concentrate ^c^	
	mg/100 g	% Total AA	mg/100 g	%Total AA	mg/100 g	%Total AA
Alanine	4322	5.6	2677	4.3	4900	5
Arginine	6115	7.9	4642	7.4	2100	2.1
Aspartic acid	6762	8.7	7249	11.5	10,800	10.9
Cystine	1629	2.1	886	1.4	2089	2.1
Glutamic acid	13,495	17.4	12,013	19.1	16,700	16.9
Glycine	3410	4.4	2688	4.3	1800	1.8
Histidine	1699	2.2	1578	2.5	2200	2.2
Isoleucine	3381	4.4	2942	4.7	5800	5.9
Leucine	6203	8	4917	7.8	10,239	10.4
Lysine	2120	2.7	3929	6.2	9600	9.7
Methionine	2279	2.9	814	1.3	1900	1.9
Phenylalanine	4116	5.3	3278	5.2	3300	3.3
Proline	3557	4.6	3298	5.2	5800	5.9
Serine	3793	4.9	3369	5.4	4700	4.8
Threonine	2799	3.6	2474	3.9	7200	7.3
Tryptophan	1120	1.4	835	1.3	2100	2.1
Tyrosine	6203	8	2301	3.7	1800	1.8
Valine	4469	5.8	3064	4.9	5800	5.9
Total amino acids	77,472	100	62,954	100	98,828	100
Total EAA/% total	28,186	36.4	23,832	37.9	52,490	53.1
Total BCAA/% total	14,053	18.1	10,923	17.4	24,100	24.4

^a^ Results of Covance^®^ Laboratories Inc. Axiom Foods Organic Oryzatein Ultra 80 Batch HZN11018-22. Sample: 1250431. Certificate of Analysis. Report 535689-0. Received on 21 March 2012. ^b^ U.S. Department of Agriculture, Agricultural Research Service. 2013. USDA National Nutrient Database for Standard Reference, Release 26, item 16420 [10]. ^c^ US Dairy Export Council. 2004. Applications Monograph. Senior Nutrition [11].

## 4. Conclusions

These results provide a valuable addition to the nutrient database of protein isolates and concentrates from cereal grains. Oryzatein-80, an organic brown rice protein concentrate or Oryzatein-90, an organic brown rice protein isolate, may be used in place of other protein isolates or concentrates without any loss of essential nutrient value.
